# 1,1-Dimethyl­biguanidium(2+) dinitrate

**DOI:** 10.1107/S1600536811051105

**Published:** 2011-12-03

**Authors:** Michaela Fridrichová, Ivana Císařová, Ivan Němec

**Affiliations:** a Department of Inorganic Chemistry, Faculty of Science, Charles University in Prague, Hlavova 2030, 12843 Prague 2, Czech Republic

## Abstract

In the crystal structure of the title compound, C_4_H_13_N_5_^2+^·2NO_3_^−^, the main inter­molecular inter­actions are the N—H⋯O hydrogen bonds between the cationic amino groups and the O atoms of the nitrate ions. All amino H atoms and nitrate O atoms are involved in the three-dimensional hydrogen-bond network. There are two graph-set motifs *R*_2_^2^(8), which include the amino groups connected to the N atoms in the biguanide 3-, 4- and 5-positions, and the O atoms of a nitrate ion. They are extended along the *a* axis. An O atom of the second nitrate ion is involved in a graph-set motif *C*(4) that is a part of a helix-like N—H⋯O⋯H—N—H⋯O⋯ chain oriented along the *b* axis. There are also two weak C—H⋯O inter­actions in the crystal structure.

## Related literature

For uses of biguanide derivatives in medicine, see: Watkins *et al.* (1987 ▶). For applications of 1,1-dimethyl­biguanide, see: Bell & Hadden (1997 ▶); Hopker (1961 ▶); Wiernsperger (2000 ▶). For 1,1-dimethyl­biguanide in metal complexes, see: Gheorghiu (1969 ▶); Marchi *et al.* (1999 ▶); Spacu & Gheorghiu (1968 ▶, 1969 ▶); Viossat *et al.* (1995 ▶); Zhu *et al.* (2002 ▶). For related structures of monocation salts, see: Hariharan *et al.* (1989 ▶); He *et al.* (2002 ▶); Huang *et al.* (2008 ▶); Lu *et al.* (2004*a* ▶); Zhu *et al.* (2003 ▶). For related structures of dication salts, see: Lemoine *et al.* (1994 ▶); Lu *et al.* (2004*b* ▶). For related salt materials, see: Fridrichová *et al.* (2010 ▶); Matulková *et al.* (2011 ▶). For graph-set analysis of hydrogen bonds, see: Etter *et al.* (1990 ▶). For details of the Cambridge Structural Database, see: Allen (2002 ▶).
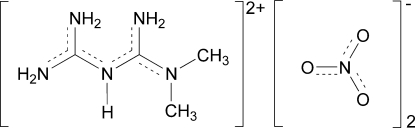


## Experimental

### 

#### Crystal data

C_4_H_13_N_5_^2+^·2NO_3_^−^
*M* *_r_* = 255.21
Monoclinic, 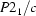

*a* = 7.7850 (2) Å
*b* = 5.7313 (2) Å
*c* = 26.5321 (7) Å
β = 101.6020 (15)°
*V* = 1159.63 (6) Å^3^
*Z* = 4
Mo *K*α radiation
μ = 0.13 mm^−1^
*T* = 150 K
0.4 × 0.3 × 0.18 mm

#### Data collection

Nonius KappaCCD area-detector diffractometer
13222 measured reflections
2228 independent reflections
1913 reflections with *I* > 2σ(*I*)
*R*_int_ = 0.050

#### Refinement

*R*[*F* ^2^ > 2σ(*F* ^2^)] = 0.041
*wR*(*F* ^2^) = 0.113
*S* = 1.08
2228 reflections
178 parameters
7 restraints
H atoms treated by a mixture of independent and constrained refinement
Δρ_max_ = 0.19 e Å^−3^
Δρ_min_ = −0.24 e Å^−3^

### 

Data collection: *COLLECT* (Hooft, 1998 ▶) and *DENZO* (Otwinowski & Minor, 1997 ▶); cell refinement: *COLLECT* and *DENZO*; data reduction: *COLLECT* and *DENZO*; program(s) used to solve structure: *SIR92* (Altomare *et al.*, 1993 ▶); program(s) used to refine structure: *SHELXL97* (Sheldrick, 2008 ▶); molecular graphics: *PLATON* (Spek, 2009 ▶) and *DIAMOND* (Brandenburg, 2006 ▶); software used to prepare material for publication: *publCIF* (Westrip, 2010 ▶).

## Supplementary Material

Crystal structure: contains datablock(s) global, I. DOI: 10.1107/S1600536811051105/zq2139sup1.cif

Structure factors: contains datablock(s) I. DOI: 10.1107/S1600536811051105/zq2139Isup2.hkl

Supplementary material file. DOI: 10.1107/S1600536811051105/zq2139Isup3.smi

Additional supplementary materials:  crystallographic information; 3D view; checkCIF report

## Figures and Tables

**Table 1:** Hydrogen-bond geometry (Å, °)

*D*—H⋯*A*	*D*—H	H⋯*A*	*D*⋯*A*	*D*—H⋯*A*
N1—H12⋯O2	0.89 (1)	2.06 (2)	2.9158 (17)	160 (2)
N1—H12⋯O1	0.89 (1)	2.57 (2)	3.3151 (17)	142 (1)
N1—H11⋯O5	0.88 (1)	2.09 (2)	2.9463 (16)	164 (2)
N1—H11⋯O4	0.88 (1)	2.57 (2)	3.2920 (17)	140 (1)
N2—H21⋯O4	0.84 (1)	2.15 (2)	2.9571 (18)	161 (2)
N2—H21⋯O4^i^	0.84 (1)	2.56 (2)	3.0833 (17)	122 (2)
N2—H22⋯O6^ii^	0.87 (1)	2.15 (2)	2.9674 (18)	157 (2)
N2—H22⋯O6^i^	0.87 (1)	2.64 (2)	3.2503 (18)	128 (2)
N3—H3⋯O5^ii^	0.85 (1)	2.05 (2)	2.8479 (16)	157 (2)
N3—H3⋯O6^ii^	0.85 (1)	2.59 (2)	3.2947 (17)	142 (2)
N4—H42⋯O3^iii^	0.85 (1)	2.17 (2)	2.9300 (18)	149 (2)
N4—H42⋯O1^iii^	0.85 (1)	2.34 (2)	3.1164 (18)	153 (2)
N4—H41⋯O3^iv^	0.89 (1)	1.97 (2)	2.8487 (17)	170 (2)
C3—H3*B*⋯O2^ii^	0.98	2.42	3.255 (2)	143
C4—H4*A*⋯O6^v^	0.98	2.55	3.519 (2)	170

Symmetry codes: (i) 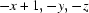; (ii) 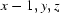; (iii) 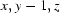; (iv) 
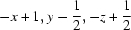
; (v) 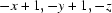.

**Fig. 1.**
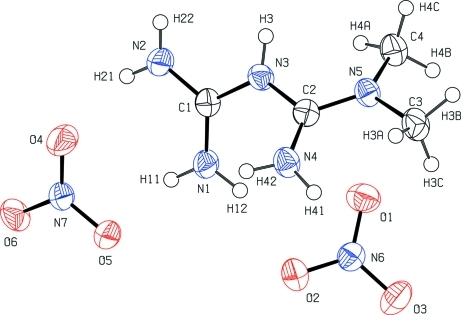
Atom-labelling scheme of the title compound. Displacement ellipsoids are drawn at the 50% probability level.

**Fig. 2.**
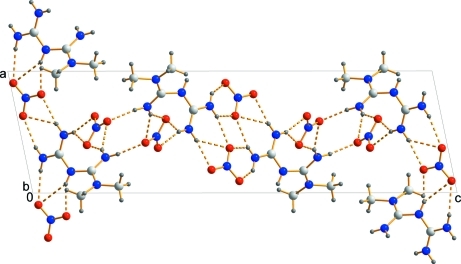
Packing scheme of the title compound along the monoclinic axis (blue: N; red: O; large grey spheres: C; small grey spheres: H). The dashed lines indicate the hydrogen bonds.

**Fig. 3.**
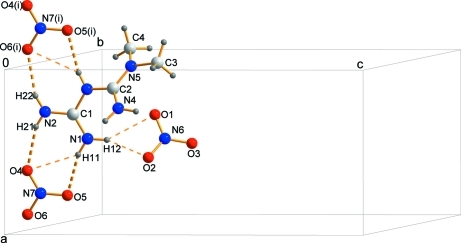
Section of the structure showing the graph-set motifs R22(8) extended along the a axis. The hydrogen bonds involved in these graph-set motifs are enhanced (blue: N; red: O; large grey spheres: C; small grey spheres: H; symmetry code (i) = 1 - x, y, z).

**Fig. 4.**
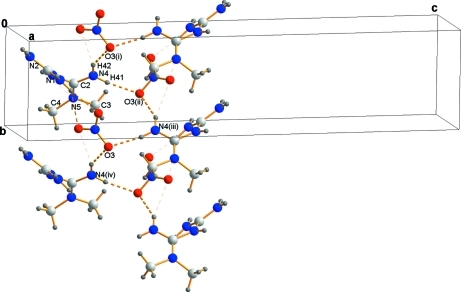
View of the graph set motif C(4). The atoms involved are H42—N4—H41···O3(ii)···H42—N4(iii) etc. The hydrogen bonds pertinent to the C(4) motif are enhanced (blue: N; red: O; large grey spheres: C; small grey spheres: H; symmetry codes (i) = x, y - 1, z; (ii) = 1 - x, -1/2 + y, 1/2 - z; (iii) = 1 - x, 1/2 + y, 1/2 - z; (iv) = x, y + 1, z).

## supplementary crystallographic information

### Comment

Among derivatives of biguanide widely used in medicine (Watkins *et al.*, 1987), 1,1-dimethylbiguanide, known also under common name *metformin*, is an important component of drugs for diabetes treatment (Hopker, 1961; Wiernsperger, 2000). An overview of the investigation and use of 1,1-dimethylbiguanide has been published by Bell & Hadden (1997).

Complexes of 1,1-dimethylbiguanide, namely Cu(II) (Viossat *et al.*, 1995; Zhu *et al.*, 2002), Rh(III) (Spacu & Gheorghiu, 1968), Ir(III) (Gheorghiu, 1969), Os(II) and Os(III) (Spacu & Gheorghiu, 1969), Tc(V) and Re(V) complexes (Marchi *et al.*, 1999) have been studied.

Surprisingly only eight structures concerning 1,1-dimethylbiguanide are available in the current version of the Cambridge Structure Database (Allen, 2002). Five of them contain a monocation: chloride (Hariharan *et al.*, 1989), bromide (Lu *et al.*, 2004*a*), nitrate (Zhu *et al.*, 2003), [TlBr_4_]^3-^ salt (He *et al.*, 2002) and perchlorate, with the cation enclosed in a large complex (Huang *et al.*, 2008). Other three salts contain dications: oxalate (Lu *et al.*, 2004*b*), sulfate (Lu *et al.*, 2004*b*) and a [CuCl_4_]^2-^ complex (Lemoine *et al.*, 1994).

In the crystal structure of the title compound, 1,1-Dimethylbiguanidium(2+) dinitrate, the main intermolecular interactions are the N—H···O hydrogen bonds between the amino groups of the cations and the O atoms of the nitrate anions with distances ranging from 2.848 (2) to 3.315 (2) Å, forming a three dimensional network (Fig. 2). Along the *a* axis there are extended two graph set motifs *R*^2^_2_(8) (Etter *et al.*, 1990; Fig. 3). The atoms involved in these two graph set motifs are N7—O5···H11—N1—C1—N2—H21···O4-(N7) and C1—N3—H3···O5(i)-N7(i)-O6(i)···H22—N22-(C1) with the symmetry code (i) = 1 - *x*, *y*, *z*. Along the *b* axis there is a chain O3(i)···H42—N4—H41···O3(ii)··· (Fig. 4; symmetry codes (i) = *x*, *y* - 1, *z*; (ii) = 1 - *x*, -1/2 + *y*, 1/2 - *z*) containing the graph set motif C(4). This chain forms a helix with the axis parallel to *b*. Two consecutive graph set motifs C(4) correspond to the pitch of this helix which equals to the unit-cell length *b*. O1 and O2 are involved in N1—H12···O1 and N1—H12···O2, respectively. In comparison to the monocation nitrate (Zhu *et al.*, 2003), there are no important hydrogen bonds between the cations.

The title compound was prepared during the primary research oriented on salt materials with delocalized π electrons for applications in non-linear optics. For comparison, see similar salt materials (Fridrichová *et al.*, 2010; Matulková *et al.*, 2011).

### Experimental

A solution of the title compound was prepared by neutralization of stoichiometric amounts of dimethylbiguanidium hydroxide and nitric acid. The hydroxide was prepared from dimethylbiguanide hydrochloride (97%, Sigma-Aldrich) by the exchange reaction on an anex. Small transparent colourless crystals were obtained from water solution first after two years of crystallization. They were stable on air and non-hygroscopic.

### Refinement

The primary amino H atoms were located in a difference Fourier map and refined with a N—H distance restraint equal to 0.88 Å and with *U*_iso_(H) = 1.2*U*_eq_(N). All other H positions were calculated after each cycle of refinement using a riding model, with C—H = 0.98 Å for methyl H atoms and *U*_iso_(H) = 1.5*U*_eq_(C).

### Crystal data

**Table:**

C_4_H_13_N_5_^2+^·2NO_3_^−^	*F*(000) = 536
*M**_r_* = 255.21	*D*_x_ = 1.462 Mg m^−^^3^
Monoclinic, *P*2_1_/*c*	Mo *K*α radiation, λ = 0.71073 Å
Hall symbol: -P 2ybc	Cell parameters from 2377 reflections
*a* = 7.7850 (2) Å	θ = 1–26.0°
*b* = 5.7313 (2) Å	µ = 0.13 mm^−^^1^
*c* = 26.5321 (7) Å	*T* = 150 K
β = 101.6020 (15)°	Plate, colourless
*V* = 1159.63 (6) Å^3^	0.4 × 0.3 × 0.18 mm
*Z* = 4	

### Data collection

**Table:**

Nonius KappaCCD area-detector diffractometer	1913 reflections with *I* > 2σ(*I*)
Radiation source: fine-focus sealed tube	*R*_int_ = 0.050
graphite	θ_max_ = 26.0°, θ_min_ = 2.7°
Detector resolution: 9.091 pixels mm^-1^	*h* = −9→9
φ and ω scans to fill the Ewald sphere	*k* = −7→7
13222 measured reflections	*l* = −32→32
2228 independent reflections	

### Refinement

**Table:**

Refinement on *F*^2^	Primary atom site location: structure-invariant direct methods
Least-squares matrix: full	Secondary atom site location: difference Fourier map
*R*[*F*^2^ > 2σ(*F*^2^)] = 0.041	Hydrogen site location: difference Fourier map
*wR*(*F*^2^) = 0.113	H atoms treated by a mixture of independent and constrained refinement
*S* = 1.08	*w* = 1/[σ^2^(*F*_o_^2^) + (0.0581*P*)^2^ + 0.3152*P*] where *P* = (*F*_o_^2^ + 2*F*_c_^2^)/3
2228 reflections	(Δ/σ)_max_ < 0.001
178 parameters	Δρ_max_ = 0.19 e Å^−^^3^
7 restraints	Δρ_min_ = −0.24 e Å^−^^3^

### Special details

**Table:**

Experimental. Vibrational spectra were recorded on a Nicolet Magna 760 FTIR spectrometer: IR spectra using DRIFTS technique in the 100 - 4000 cm^-1^ region, with 2 cm^-1^ resolution and Happ-Genzel apodization, Raman spectra using Nicolet Nexus FT Raman module (1064 nm N d:YVO4 laser excitation, 200 mW power at the sample) in the 100–3700 cm^-1^ region, with 2 cm^-1^ resolution and Happ-Genzel apodization. For further characterization of the studied compound vibrational spectra are given as a peaklist: IR spectra: 464 m, 505 m, 583 m, 602 m, 722 m, 824 m, 848 w, 937 m, 981 m,1043 m, 1052 m, 1111 m, 1145 m, 1184 m, 1283 m, 1312 m, 1385 m, 1406 s, 1416 s, 1459 m,1486 s, 1574 s, 1600 s, 1630 s, 1659 s, 1748 w, 2080 w, 2134 w, 2340 w, 2445 w, 2471 w, 2619 w, 2737 w, 2797 w, 2879 m, 2953 m, 2981 m, 3177 s, 3327 s. Raman spectra: 158 s, 197 m, 258 w, 332 w, 409 w, 441 w, 476 w, 490 w, 602 m, 709 w, 736 m, 844 m, 939 s, 1042 versus, 1061 m, 1100 m, 1144 w, 1186 w, 1262 w, 1286 w, 1413 m, 1430 w, 1457 m, 1475 m, 1500 m, 1597 w, 1662 w, 1674 w, 2813 w, 2880 m, 2935 s, 2956 m, 3012 w, 3114 w,3220 w, 3294 w, 3340 w.
Geometry. All e.s.d.'s (except the e.s.d. in the dihedral angle between two l.s. planes) are estimated using the full covariance matrix. The cell e.s.d.'s are taken into account individually in the estimation of e.s.d.'s in distances, angles and torsion angles; correlations between e.s.d.'s in cell parameters are only used when they are defined by crystal symmetry. An approximate (isotropic) treatment of cell e.s.d.'s is used for estimating e.s.d.'s involving l.s. planes.
Refinement. Refinement of *F*^2^ against ALL reflections. The weighted *R*-factor *wR* and goodness of fit *S* are based on *F*^2^, conventional *R*-factors *R* are based on *F*, with *F* set to zero for negative *F*^2^. The threshold expression of *F*^2^ > σ(*F*^2^) is used only for calculating *R*-factors(gt) *etc*. and is not relevant to the choice of reflections for refinement. *R*-factors based on *F*^2^ are statistically about twice as large as those based on *F*, and *R*- factors based on ALL data will be even larger.

### Fractional atomic coordinates and isotropic or equivalent isotropic displacement parameters (Å^2^)

**Table:**

	*x*	*y*	*z*	*U*_iso_*/*U*_eq_	
C1	0.31918 (18)	0.4058 (2)	0.08102 (5)	0.0302 (3)	
C2	0.18235 (17)	0.5649 (2)	0.14924 (5)	0.0292 (3)	
C3	0.0566 (2)	0.8084 (3)	0.20625 (6)	0.0413 (4)	
H3A	0.0727	0.6757	0.2300	0.062*	
H3B	−0.0587	0.8789	0.2053	0.062*	
H3C	0.1484	0.9245	0.2179	0.062*	
C4	−0.0215 (2)	0.8737 (3)	0.11244 (6)	0.0390 (4)	
H4A	0.0252	0.8402	0.0815	0.059*	
H4B	−0.0020	1.0386	0.1217	0.059*	
H4C	−0.1475	0.8403	0.1056	0.059*	
N1	0.47682 (16)	0.4787 (2)	0.10136 (5)	0.0346 (3)	
H11	0.567 (2)	0.428 (3)	0.0891 (7)	0.042*	
H12	0.497 (2)	0.586 (3)	0.1260 (6)	0.042*	
N2	0.28755 (18)	0.2669 (2)	0.04101 (5)	0.0356 (3)	
H21	0.372 (2)	0.217 (3)	0.0291 (7)	0.043*	
H22	0.184 (2)	0.210 (3)	0.0295 (7)	0.043*	
N3	0.17655 (15)	0.4775 (2)	0.10035 (4)	0.0312 (3)	
H3	0.0744 (19)	0.459 (3)	0.0822 (6)	0.037*	
N4	0.29198 (17)	0.4743 (2)	0.18829 (5)	0.0349 (3)	
H41	0.319 (2)	0.542 (3)	0.2192 (6)	0.042*	
H42	0.354 (2)	0.357 (3)	0.1842 (7)	0.042*	
N5	0.06775 (15)	0.7280 (2)	0.15485 (4)	0.0315 (3)	
N6	0.53721 (16)	0.9772 (2)	0.18120 (4)	0.0338 (3)	
O1	0.39367 (14)	0.9879 (2)	0.15067 (4)	0.0453 (3)	
O2	0.62993 (15)	0.7999 (2)	0.18337 (5)	0.0505 (4)	
O3	0.58703 (17)	1.1475 (2)	0.21013 (4)	0.0495 (3)	
N7	0.78091 (15)	0.2624 (2)	0.02958 (5)	0.0327 (3)	
O4	0.63326 (15)	0.1814 (2)	0.01442 (5)	0.0581 (4)	
O5	0.81006 (13)	0.3970 (2)	0.06767 (4)	0.0412 (3)	
O6	0.90260 (14)	0.2106 (2)	0.00741 (4)	0.0446 (3)	

### Atomic displacement parameters (Å^2^)

**Table:**

	*U*^11^	*U*^22^	*U*^33^	*U*^12^	*U*^13^	*U*^23^
C1	0.0339 (7)	0.0290 (7)	0.0289 (7)	0.0024 (6)	0.0091 (5)	0.0016 (5)
C2	0.0290 (7)	0.0307 (7)	0.0303 (7)	−0.0027 (6)	0.0119 (5)	0.0016 (5)
C3	0.0397 (8)	0.0511 (10)	0.0367 (8)	0.0086 (7)	0.0164 (6)	−0.0039 (7)
C4	0.0417 (8)	0.0349 (8)	0.0411 (8)	0.0073 (7)	0.0099 (6)	0.0040 (6)
N1	0.0315 (7)	0.0381 (7)	0.0371 (7)	−0.0009 (5)	0.0137 (5)	−0.0086 (5)
N2	0.0357 (7)	0.0412 (7)	0.0308 (6)	0.0008 (6)	0.0087 (5)	−0.0071 (5)
N3	0.0273 (6)	0.0373 (7)	0.0298 (6)	0.0026 (5)	0.0074 (5)	−0.0018 (5)
N4	0.0381 (7)	0.0377 (7)	0.0302 (6)	0.0078 (6)	0.0095 (5)	0.0022 (5)
N5	0.0306 (6)	0.0344 (6)	0.0318 (6)	0.0033 (5)	0.0116 (5)	0.0007 (5)
N6	0.0361 (7)	0.0345 (7)	0.0321 (6)	−0.0019 (5)	0.0098 (5)	−0.0009 (5)
O1	0.0395 (6)	0.0408 (7)	0.0513 (7)	−0.0015 (5)	−0.0010 (5)	0.0057 (5)
O2	0.0410 (7)	0.0419 (7)	0.0667 (8)	0.0099 (5)	0.0062 (5)	−0.0108 (6)
O3	0.0708 (8)	0.0346 (6)	0.0387 (6)	−0.0015 (6)	0.0004 (5)	−0.0067 (5)
N7	0.0311 (6)	0.0348 (7)	0.0337 (6)	0.0022 (5)	0.0100 (5)	−0.0032 (5)
O4	0.0355 (6)	0.0671 (8)	0.0749 (9)	−0.0132 (6)	0.0188 (6)	−0.0362 (7)
O5	0.0373 (6)	0.0458 (7)	0.0400 (6)	0.0018 (5)	0.0069 (4)	−0.0150 (5)
O6	0.0345 (6)	0.0628 (8)	0.0403 (6)	0.0094 (5)	0.0163 (5)	−0.0049 (5)

### Geometric parameters (Å, °)

**Table:**

C1—N1	1.3060 (19)	N1—H11	0.878 (14)
C1—N2	1.3100 (19)	N1—H12	0.891 (14)
C1—N3	1.3763 (17)	N2—H21	0.837 (14)
C2—N4	1.3098 (18)	N2—H22	0.867 (14)
C2—N5	1.3212 (18)	N3—H3	0.849 (14)
C2—N3	1.3827 (17)	N4—H41	0.893 (14)
C3—N5	1.4584 (19)	N4—H42	0.846 (14)
C3—H3A	0.9800	N6—O2	1.2411 (17)
C3—H3B	0.9800	N6—O1	1.2428 (16)
C3—H3C	0.9800	N6—O3	1.2538 (16)
C4—N5	1.4601 (19)	N7—O4	1.2301 (16)
C4—H4A	0.9800	N7—O6	1.2474 (15)
C4—H4B	0.9800	N7—O5	1.2551 (16)
C4—H4C	0.9800		
			
N1—C1—N2	122.49 (13)	H11—N1—H12	117.7 (16)
N1—C1—N3	120.79 (13)	C1—N2—H21	118.6 (12)
N2—C1—N3	116.70 (13)	C1—N2—H22	121.5 (12)
N4—C2—N5	122.59 (13)	H21—N2—H22	119.3 (18)
N4—C2—N3	119.46 (13)	C1—N3—C2	125.54 (12)
N5—C2—N3	117.80 (12)	C1—N3—H3	119.0 (12)
N5—C3—H3A	109.5	C2—N3—H3	115.3 (12)
N5—C3—H3B	109.5	C2—N4—H41	123.6 (12)
H3A—C3—H3B	109.5	C2—N4—H42	120.7 (12)
N5—C3—H3C	109.5	H41—N4—H42	115.1 (16)
H3A—C3—H3C	109.5	C2—N5—C3	119.81 (12)
H3B—C3—H3C	109.5	C2—N5—C4	123.05 (12)
N5—C4—H4A	109.5	C3—N5—C4	115.56 (12)
N5—C4—H4B	109.5	O2—N6—O1	120.59 (12)
H4A—C4—H4B	109.5	O2—N6—O3	120.23 (12)
N5—C4—H4C	109.5	O1—N6—O3	119.18 (13)
H4A—C4—H4C	109.5	O4—N7—O6	120.27 (12)
H4B—C4—H4C	109.5	O4—N7—O5	120.08 (11)
C1—N1—H11	119.7 (11)	O6—N7—O5	119.65 (12)
C1—N1—H12	122.4 (11)		

### Hydrogen-bond geometry (Å, °)

**Table:**

*D*—H···*A*	*D*—H	H···*A*	*D*···*A*	*D*—H···*A*
N1—H12···O2	0.89 (1)	2.06 (2)	2.9158 (17)	160.(2)
N1—H12···O1	0.89 (1)	2.57 (2)	3.3151 (17)	142.(1)
N1—H11···O5	0.88 (1)	2.09 (2)	2.9463 (16)	164.(2)
N1—H11···O4	0.88 (1)	2.57 (2)	3.2920 (17)	140.(1)
N2—H21···O4	0.84 (1)	2.15 (2)	2.9571 (18)	161.(2)
N2—H21···O4^i^	0.84 (1)	2.56 (2)	3.0833 (17)	122.(2)
N2—H22···O6^ii^	0.87 (1)	2.15 (2)	2.9674 (18)	157.(2)
N2—H22···O6^i^	0.87 (1)	2.64 (2)	3.2503 (18)	128.(2)
N3—H3···O5^ii^	0.85 (1)	2.05 (2)	2.8479 (16)	157.(2)
N3—H3···O6^ii^	0.85 (1)	2.59 (2)	3.2947 (17)	142.(2)
N4—H42···O3^iii^	0.85 (1)	2.17 (2)	2.9300 (18)	149.(2)
N4—H42···O1^iii^	0.85 (1)	2.34 (2)	3.1164 (18)	153.(2)
N4—H41···O3^iv^	0.89 (1)	1.97 (2)	2.8487 (17)	170.(2)
C3—H3B···O2^ii^	0.98	2.42	3.255 (2)	143
C4—H4A···O6^v^	0.98	2.55	3.519 (2)	170

Symmetry codes: (i) −*x*+1, −*y*, −*z*; (ii) *x*−1, *y*, *z*; (iii) *x*, *y*−1, *z*; (iv) −*x*+1, *y*−1/2, −*z*+1/2; (v) −*x*+1, −*y*+1, −*z*.
